# Supplementary material for: Comparative genomics of 16 Microbacterium spp. that tolerate multiple heavy metals and antibiotics
Source: PeerJ. 2019 Jan 14;6:e6258. doi: 10.7717/peerj.6258 (PMC6336093; doi:10.7717/peerj.6258)
Supplement: Supplemental Information 2 [file peerj-07-6258-s002.docx]

**Table S1. Annotation data**

| **Isolate** | **Genes total number** | **Protein coding genes** | **Protein coding genes with function prediction** | **Protein coding genes without function prediction** | **COG clusters** | **KOG clusters** | **Pfam clusters** | **TIGRfam clusters** | **IMG Genome ID** |
| --- | --- | --- | --- | --- | --- | --- | --- | --- | --- |
| *Microbacterium* sp. A20 | 3857 | 3802 | 3019 | 783 | 1339 | 468 | 1738 | 809 | 2744055082 |
| *Microbacterium* sp. K19 | 3813 | 3758 | 3072 | 686 | 1333 | 474 | 1719 | 802 | 2744055081 |
| *Microbacterium* sp. K21 | 3709 | 3653 | 2998 | 655 | 1335 | 465 | 1731 | 802 | 2744055080 |
| *Microbacterium* sp. K22 | 3884 | 3829 | 3030 | 799 | 1333 | 466 | 1739 | 809 | 2744055079 |
| *Microbacterium* sp. K24 | 4258 | 4203 | 3334 | 869 | 1362 | 488 | 1779 | 821 | 2744055078 |
| *Microbacterium* sp. K27 | 3615 | 3559 | 2928 | 631 | 1332 | 467 | 1721 | 803 | 2744055076 |
| *Microbacterium* sp. K2B2 | 3864 | 3809 | 3022 | 787 | 1336 | 468 | 1739 | 810 | 2744055077 |
| *Microbacterium* sp. K30 | 4027 | 3972 | 3276 | 696 | 1364 | 489 | 1782 | 828 | 2744055075 |
| *Microbacterium* sp. K31 | 3643 | 3587 | 2931 | 656 | 1335 | 466 | 1723 | 805 | 2744055074 |
| *Microbacterium* sp. K33 | 3860 | 3805 | 3090 | 715 | 1326 | 471 | 1720 | 801 | 2744055073 |
| *Microbacterium* sp. K35 | 3759 | 3702 | 2920 | 782 | 1272 | 456 | 1674 | 778 | 2744055072 |
| *Microbacterium* sp*.* K36 | 3288 | 3231 | 2625 | 606 | 1267 | 457 | 1651 | 779 | 2744055071 |
| *Microbacterium* sp. K40 | 3658 | 3602 | 2944 | 658 | 1320 | 463 | 1722 | 803 | 2744055070 |
| *Microbacterium* sp. K41 | 3820 | 3762 | 2942 | 820 | 1271 | 456 | 1702 | 783 | 2744055069 |
| *Microbacterium* sp. K5D | 3741 | 3684 | 2965 | 719 | 1322 | 465 | 1735 | 806 | 2744055068 |
| *Microbacterium* sp. PF5 | 3736 | 3678 | 2958 | 720 | 1298 | 463 | 1718 | 796 | 2744055067 |
