# Supplementary material for: Comparative genomics of 16 Microbacterium spp. that tolerate multiple heavy metals and antibiotics
Source: PeerJ. 2019 Jan 14;6:e6258. doi: 10.7717/peerj.6258 (PMC6336093; doi:10.7717/peerj.6258)
Supplement: Supplemental Information 3 [file peerj-07-6258-s003.docx]

**Table SX2. ANI pairwise comparison**

| **Isolate** | **ANI1->2** | **ANI2->1** |
| --- | --- | --- |
| *Microbacterium* sp. A20* | 100 | 100 |
| *Microbacterium* sp. K19 | 91.67 | 91.66 |
| *Microbacterium* sp. K21 | 86.22 | 86.21 |
| *Microbacterium* sp. K22 | 99.99 | 99.99 |
| *Microbacterium* sp. K24 | 84.38 | 84.38 |
| *Microbacterium* sp. K27 | 86.29 | 86.28 |
| *Microbacterium* sp. K2B2 | 100 | 100 |
| *Microbacterium* sp. K30 | 84.06 | 84.07 |
| *Microbacterium* sp. K31 | 86.29 | 86.28 |
| *Microbacterium* sp. K33 | 91.7 | 91.69 |
| *Microbacterium* sp. K35 | 84.15 | 84.14 |
| *Microbacterium* sp. K36 | 83.42 | 83.42 |
| *Microbacterium* sp. K40 | 86.28 | 86.28 |
| *Microbacterium* sp. K41 | 84.09 | 84.11 |
| *Microbacterium* sp. K5D | 86.28 | 86.27 |
| *Microbacterium* sp. PF5 | 83.9 | 83.92 |
| *All isolates were compared to *M*. sp. A20 | | |
