# Supplementary material for: Comparative genomics of 16 Microbacterium spp. that tolerate multiple heavy metals and antibiotics
Source: PeerJ. 2019 Jan 14;6:e6258. doi: 10.7717/peerj.6258 (PMC6336093; doi:10.7717/peerj.6258)
Supplement: Supplemental Information 4 — MIC (μg/ml) of ampicillin, chloramphenicol, and vancomycin for each isolate. [file peerj-07-6258-s004.docx]

**Table S3. BLASTP comparison of the chromate reductase, ChrR**

| Genome Name | Subject Length | Gene Id | Identities | E-value |
| --- | --- | --- | --- | --- |
| *Microbacterium* sp. A20 | 194 | 2746848817 | 43% (77/179) | 3.00E-45 |
| *Microbacterium* sp. K19 | 194 | 2746844835 | 42% (76/179) | 1.00E-44 |
| *Microbacterium* sp. K21 | 194 | 2746839443 | 41% (73/179) | 1.00E-43 |
| *Microbacterium* sp. K22 | 194 | 2746839262 | 43% (77/179) | 3.00E-45 |
| *Microbacterium* sp. K24 | 188 | 2746834734 | 44% (79/179) | 3.00E-45 |
| *Microbacterium* sp. K24 | 187 | 2746831576 | 44% (78/178) | 2.00E-43 |
| *Microbacterium* sp. K27 | 194 | 2746826664 | 41% (73/180) | 7.00E-44 |
| *Microbacterium* sp. K2B2 | 194 | 2746830589 | 43% (77/179) | 3.00E-45 |
| *Microbacterium* sp. K30 | 188 | 2746822510 | 46% (77/168) | 4.00E-44 |
| *Microbacterium* sp. K30 | 193 | 2746821088 | 42% (74/178) | 4.00E-40 |
| *Microbacterium* sp. K31 | 194 | 2746817949 | 41% (73/180) | 7.00E-44 |
| *Microbacterium* sp*.* K33 | 194 | 2746815498 | 42% (76/179) | 1.00E-44 |
| *Microbacterium* sp*.* K35 | 193 | 2746808758 | 42% (74/178) | 5.00E-45 |
| *Microbacterium* sp*.* K36 | 193 | 2746808405 | 41% (74/180) | 5.00E-43 |
| *Microbacterium* sp*.* K40 | 194 | 2746801872 | 40% (72/179) | 8.00E-43 |
| *Microbacterium* sp*.* K41 | 193 | 2746798660 | 42% (74/178) | 2.00E-45 |
| *Microbacterium* sp*.* K5D | 194 | 2746794194 | 40% (72/179) | 8.00E-43 |
| *Microbacterium* sp*.* PF5 | 193 | 2746791861 | 42% (74/178) | 5.00E-45 |
| *Microbacterium* sp*.* Cr-K1W | 194 | [2529446915](https://img.jgi.doe.gov/cgi-bin/mer/main.cgi?section=GeneDetail&page=geneDetail&gene_oid=2529446915) | 42% (76/179) | 1.00E-44 |
| *Microbacterium* sp*.* Cr-K20 | 194 | [2529451669](https://img.jgi.doe.gov/cgi-bin/mer/main.cgi?section=GeneDetail&page=geneDetail&gene_oid=2529451669) | 42% (76/179) | 1.00E-44 |
| *Microbacterium* sp*.* Cr-K29 | 194 | 2529452855 | 40% (72/179) | 8.00E-43 |
| *Microbacterium* sp*.* Cr-K32 | 194 | [2529440805](https://img.jgi.doe.gov/cgi-bin/mer/main.cgi?section=GeneDetail&page=geneDetail&gene_oid=2529440805) | 41% (73/179) | 1.00E-43 |
| *Pseudomonas* *putida* KT2440 | 186 | [2667648199](https://img.jgi.doe.gov/cgi-bin/mer/main.cgi?section=GeneDetail&page=geneDetail&gene_oid=2667648199) | 100% (186/186) | 2.00E-138 |
