# Supplementary material for: Comparative genomics of 16 Microbacterium spp. that tolerate multiple heavy metals and antibiotics
Source: PeerJ. 2019 Jan 14;6:e6258. doi: 10.7717/peerj.6258 (PMC6336093; doi:10.7717/peerj.6258)
Supplement: Supplemental Information 5 [file peerj-07-6258-s005.docx]

**Table S4. Antibiotic and metal tolerance genes defined by KEGG terms or COG categories**

**Isolates**

| **Function ID** | [**Name**](https://img.jgi.doe.gov/cgi-bin/mer/yui-dt0-href-Name) | [**KEGG Pathways via KO Terms**](https://img.jgi.doe.gov/cgi-bin/mer/yui-dt0-href-MicspA202744055082) | [**A20**](https://img.jgi.doe.gov/cgi-bin/mer/yui-dt0-href-MicspK192744055081) | [**K19**](https://img.jgi.doe.gov/cgi-bin/mer/yui-dt0-href-MicspK212744055080) | [**K21**](https://img.jgi.doe.gov/cgi-bin/mer/yui-dt0-href-MicspK222744055079) | | [**K22**](https://img.jgi.doe.gov/cgi-bin/mer/yui-dt0-href-MicspK242744055078) | | [**K24**](https://img.jgi.doe.gov/cgi-bin/mer/yui-dt0-href-MicspK272744055076) | | [**K27**](https://img.jgi.doe.gov/cgi-bin/mer/yui-dt0-href-MicspK222744055077) | | [**K22**](https://img.jgi.doe.gov/cgi-bin/mer/yui-dt0-href-MicspK302744055075) | | [**K30**](https://img.jgi.doe.gov/cgi-bin/mer/yui-dt0-href-MicspK312744055074) | | [**K31**](https://img.jgi.doe.gov/cgi-bin/mer/yui-dt0-href-MicspK332744055073) | | [**K33**](https://img.jgi.doe.gov/cgi-bin/mer/yui-dt0-href-MicspK352744055072) | | [**K35**](https://img.jgi.doe.gov/cgi-bin/mer/yui-dt0-href-MicspK362744055071) | | [**K36**](https://img.jgi.doe.gov/cgi-bin/mer/yui-dt0-href-MicspK402744055070) | | [**K40**](https://img.jgi.doe.gov/cgi-bin/mer/yui-dt0-href-MicspK412744055069) | | [**K41**](https://img.jgi.doe.gov/cgi-bin/mer/yui-dt0-href-MicspK5D2744055068) | | [**K5D**](https://img.jgi.doe.gov/cgi-bin/mer/yui-dt0-href-MicspPF52744055067) | | **PF5** | |
| --- | --- | --- | --- | --- | --- | --- | --- | --- | --- | --- | --- | --- | --- | --- | --- | --- | --- | --- | --- | --- | --- | --- | --- | --- | --- | --- | --- | --- | --- | --- | --- | --- |
| KO:K17836 | beta-lactamase class A [EC:3.5.2.6] (penP) | beta-Lactam resistance | [4](https://img.jgi.doe.gov/cgi-bin/mer/yui-dt0-href-MicspK192744055081) | [4](https://img.jgi.doe.gov/cgi-bin/mer/yui-dt0-href-MicspK212744055080) | [2](https://img.jgi.doe.gov/cgi-bin/mer/yui-dt0-href-MicspK222744055079) | [4](https://img.jgi.doe.gov/cgi-bin/mer/yui-dt0-href-MicspK242744055078) | | [1](https://img.jgi.doe.gov/cgi-bin/mer/yui-dt0-href-MicspK272744055076) | | [2](https://img.jgi.doe.gov/cgi-bin/mer/yui-dt0-href-MicspK222744055077) | | [4](https://img.jgi.doe.gov/cgi-bin/mer/yui-dt0-href-MicspK302744055075) | | [1](https://img.jgi.doe.gov/cgi-bin/mer/yui-dt0-href-MicspK312744055074) | | [2](https://img.jgi.doe.gov/cgi-bin/mer/yui-dt0-href-MicspK332744055073) | | [4](https://img.jgi.doe.gov/cgi-bin/mer/yui-dt0-href-MicspK352744055072) | | [3](https://img.jgi.doe.gov/cgi-bin/mer/yui-dt0-href-MicspK362744055071) | | [4](https://img.jgi.doe.gov/cgi-bin/mer/yui-dt0-href-MicspK402744055070) | | [2](https://img.jgi.doe.gov/cgi-bin/mer/yui-dt0-href-MicspK412744055069) | | [3](https://img.jgi.doe.gov/cgi-bin/mer/yui-dt0-href-MicspK5D2744055068) | | [2](https://img.jgi.doe.gov/cgi-bin/mer/yui-dt0-href-MicspPF52744055067) | | 3 | |  |
| KO:K01207 | beta-N-acetylhexosaminidase [EC:3.2.1.52] (nagZ) | beta-Lactam resistance | [2](https://img.jgi.doe.gov/cgi-bin/mer/yui-dt0-href-MicspK192744055081) | [2](https://img.jgi.doe.gov/cgi-bin/mer/yui-dt0-href-MicspK212744055080) | [2](https://img.jgi.doe.gov/cgi-bin/mer/yui-dt0-href-MicspK222744055079) | [2](https://img.jgi.doe.gov/cgi-bin/mer/yui-dt0-href-MicspK242744055078) | | [2](https://img.jgi.doe.gov/cgi-bin/mer/yui-dt0-href-MicspK272744055076) | | [2](https://img.jgi.doe.gov/cgi-bin/mer/yui-dt0-href-MicspK222744055077) | | [2](https://img.jgi.doe.gov/cgi-bin/mer/yui-dt0-href-MicspK302744055075) | | [2](https://img.jgi.doe.gov/cgi-bin/mer/yui-dt0-href-MicspK312744055074) | | [2](https://img.jgi.doe.gov/cgi-bin/mer/yui-dt0-href-MicspK332744055073) | | [2](https://img.jgi.doe.gov/cgi-bin/mer/yui-dt0-href-MicspK352744055072) | | [1](https://img.jgi.doe.gov/cgi-bin/mer/yui-dt0-href-MicspK362744055071) | | [2](https://img.jgi.doe.gov/cgi-bin/mer/yui-dt0-href-MicspK402744055070) | | [2](https://img.jgi.doe.gov/cgi-bin/mer/yui-dt0-href-MicspK412744055069) | | [1](https://img.jgi.doe.gov/cgi-bin/mer/yui-dt0-href-MicspK5D2744055068) | | [2](https://img.jgi.doe.gov/cgi-bin/mer/yui-dt0-href-MicspPF52744055067) | | 3 | |  |
| KO:K15580 | oligopeptide transport system substrate-binding protein (oppA, mppA) | beta-Lactam resistance | [1](https://img.jgi.doe.gov/cgi-bin/mer/yui-dt0-href-MicspK192744055081) | [2](https://img.jgi.doe.gov/cgi-bin/mer/yui-dt0-href-MicspK212744055080) | [1](https://img.jgi.doe.gov/cgi-bin/mer/yui-dt0-href-MicspK222744055079) | [1](https://img.jgi.doe.gov/cgi-bin/mer/yui-dt0-href-MicspK242744055078) | | [1](https://img.jgi.doe.gov/cgi-bin/mer/yui-dt0-href-MicspK272744055076) | | [1](https://img.jgi.doe.gov/cgi-bin/mer/yui-dt0-href-MicspK222744055077) | | [1](https://img.jgi.doe.gov/cgi-bin/mer/yui-dt0-href-MicspK302744055075) | | [1](https://img.jgi.doe.gov/cgi-bin/mer/yui-dt0-href-MicspK312744055074) | | [1](https://img.jgi.doe.gov/cgi-bin/mer/yui-dt0-href-MicspK332744055073) | | [2](https://img.jgi.doe.gov/cgi-bin/mer/yui-dt0-href-MicspK352744055072) | | [1](https://img.jgi.doe.gov/cgi-bin/mer/yui-dt0-href-MicspK362744055071) | | [1](https://img.jgi.doe.gov/cgi-bin/mer/yui-dt0-href-MicspK402744055070) | | [1](https://img.jgi.doe.gov/cgi-bin/mer/yui-dt0-href-MicspK412744055069) | | [1](https://img.jgi.doe.gov/cgi-bin/mer/yui-dt0-href-MicspK5D2744055068) | | [1](https://img.jgi.doe.gov/cgi-bin/mer/yui-dt0-href-MicspPF52744055067) | | 1 | |  |
| KO:K15581 | oligopeptide transport system permease protein (oppB) | beta-Lactam resistance | [1](https://img.jgi.doe.gov/cgi-bin/mer/main.cgi?section=PhyloProfile&page=phyloProfileGenes&type=func&procId=80526&id=COG0053&taxon_oid=2744055081&bin_oid=) | [2](https://img.jgi.doe.gov/cgi-bin/mer/main.cgi?section=PhyloProfile&page=phyloProfileGenes&type=func&procId=80526&id=COG0053&taxon_oid=2744055080&bin_oid=) | [1](https://img.jgi.doe.gov/cgi-bin/mer/main.cgi?section=PhyloProfile&page=phyloProfileGenes&type=func&procId=80526&id=COG0053&taxon_oid=2744055079&bin_oid=) | [1](https://img.jgi.doe.gov/cgi-bin/mer/main.cgi?section=PhyloProfile&page=phyloProfileGenes&type=func&procId=80526&id=COG0053&taxon_oid=2744055078&bin_oid=) | | [1](https://img.jgi.doe.gov/cgi-bin/mer/main.cgi?section=PhyloProfile&page=phyloProfileGenes&type=func&procId=80526&id=COG0053&taxon_oid=2744055076&bin_oid=) | | [1](https://img.jgi.doe.gov/cgi-bin/mer/main.cgi?section=PhyloProfile&page=phyloProfileGenes&type=func&procId=80526&id=COG0053&taxon_oid=2744055077&bin_oid=) | | [1](https://img.jgi.doe.gov/cgi-bin/mer/main.cgi?section=PhyloProfile&page=phyloProfileGenes&type=func&procId=80526&id=COG0053&taxon_oid=2744055075&bin_oid=) | | [1](https://img.jgi.doe.gov/cgi-bin/mer/main.cgi?section=PhyloProfile&page=phyloProfileGenes&type=func&procId=80526&id=COG0053&taxon_oid=2744055074&bin_oid=) | | [1](https://img.jgi.doe.gov/cgi-bin/mer/main.cgi?section=PhyloProfile&page=phyloProfileGenes&type=func&procId=80526&id=COG0053&taxon_oid=2744055073&bin_oid=) | | [2](https://img.jgi.doe.gov/cgi-bin/mer/main.cgi?section=PhyloProfile&page=phyloProfileGenes&type=func&procId=80526&id=COG0053&taxon_oid=2744055072&bin_oid=) | | [1](https://img.jgi.doe.gov/cgi-bin/mer/main.cgi?section=PhyloProfile&page=phyloProfileGenes&type=func&procId=80526&id=COG0053&taxon_oid=2744055071&bin_oid=) | | [1](https://img.jgi.doe.gov/cgi-bin/mer/main.cgi?section=PhyloProfile&page=phyloProfileGenes&type=func&procId=80526&id=COG0053&taxon_oid=2744055070&bin_oid=) | | [1](https://img.jgi.doe.gov/cgi-bin/mer/main.cgi?section=PhyloProfile&page=phyloProfileGenes&type=func&procId=80526&id=COG0053&taxon_oid=2744055069&bin_oid=) | | [1](https://img.jgi.doe.gov/cgi-bin/mer/main.cgi?section=PhyloProfile&page=phyloProfileGenes&type=func&procId=80526&id=COG0053&taxon_oid=2744055068&bin_oid=) | | [1](https://img.jgi.doe.gov/cgi-bin/mer/main.cgi?section=PhyloProfile&page=phyloProfileGenes&type=func&procId=80526&id=COG0053&taxon_oid=2744055067&bin_oid=) | | 1 | |  |
| KO:K15582 | oligopeptide transport system permease protein (oppC) | beta-Lactam resistance | [1](https://img.jgi.doe.gov/cgi-bin/mer/main.cgi?section=PhyloProfile&page=phyloProfileGenes&type=func&procId=80526&id=COG0431&taxon_oid=2744055081&bin_oid=) | [2](https://img.jgi.doe.gov/cgi-bin/mer/main.cgi?section=PhyloProfile&page=phyloProfileGenes&type=func&procId=80526&id=COG0431&taxon_oid=2744055080&bin_oid=) | [1](https://img.jgi.doe.gov/cgi-bin/mer/main.cgi?section=PhyloProfile&page=phyloProfileGenes&type=func&procId=80526&id=COG0431&taxon_oid=2744055079&bin_oid=) | [1](https://img.jgi.doe.gov/cgi-bin/mer/main.cgi?section=PhyloProfile&page=phyloProfileGenes&type=func&procId=80526&id=COG0431&taxon_oid=2744055078&bin_oid=) | | [1](https://img.jgi.doe.gov/cgi-bin/mer/main.cgi?section=PhyloProfile&page=phyloProfileGenes&type=func&procId=80526&id=COG0431&taxon_oid=2744055076&bin_oid=) | | [1](https://img.jgi.doe.gov/cgi-bin/mer/main.cgi?section=PhyloProfile&page=phyloProfileGenes&type=func&procId=80526&id=COG0431&taxon_oid=2744055077&bin_oid=) | | [1](https://img.jgi.doe.gov/cgi-bin/mer/main.cgi?section=PhyloProfile&page=phyloProfileGenes&type=func&procId=80526&id=COG0431&taxon_oid=2744055075&bin_oid=) | | [1](https://img.jgi.doe.gov/cgi-bin/mer/main.cgi?section=PhyloProfile&page=phyloProfileGenes&type=func&procId=80526&id=COG0431&taxon_oid=2744055074&bin_oid=) | | [1](https://img.jgi.doe.gov/cgi-bin/mer/main.cgi?section=PhyloProfile&page=phyloProfileGenes&type=func&procId=80526&id=COG0431&taxon_oid=2744055073&bin_oid=) | | [2](https://img.jgi.doe.gov/cgi-bin/mer/main.cgi?section=PhyloProfile&page=phyloProfileGenes&type=func&procId=80526&id=COG0431&taxon_oid=2744055072&bin_oid=) | | [1](https://img.jgi.doe.gov/cgi-bin/mer/main.cgi?section=PhyloProfile&page=phyloProfileGenes&type=func&procId=80526&id=COG0431&taxon_oid=2744055071&bin_oid=) | | [1](https://img.jgi.doe.gov/cgi-bin/mer/main.cgi?section=PhyloProfile&page=phyloProfileGenes&type=func&procId=80526&id=COG0431&taxon_oid=2744055070&bin_oid=) | | [1](https://img.jgi.doe.gov/cgi-bin/mer/main.cgi?section=PhyloProfile&page=phyloProfileGenes&type=func&procId=80526&id=COG0431&taxon_oid=2744055069&bin_oid=) | | [1](https://img.jgi.doe.gov/cgi-bin/mer/main.cgi?section=PhyloProfile&page=phyloProfileGenes&type=func&procId=80526&id=COG0431&taxon_oid=2744055068&bin_oid=) | | [1](https://img.jgi.doe.gov/cgi-bin/mer/main.cgi?section=PhyloProfile&page=phyloProfileGenes&type=func&procId=80526&id=COG0431&taxon_oid=2744055067&bin_oid=) | | 1 | |  |
| KO:K03587 | cell division protein FtsI (penicillin-binding protein 3) (ftsI) | beta-Lactam resistance | [1](https://img.jgi.doe.gov/cgi-bin/mer/main.cgi?section=PhyloProfile&page=phyloProfileGenes&type=func&procId=80526&id=COG1230&taxon_oid=2744055081&bin_oid=) | [1](https://img.jgi.doe.gov/cgi-bin/mer/main.cgi?section=PhyloProfile&page=phyloProfileGenes&type=func&procId=80526&id=COG1230&taxon_oid=2744055080&bin_oid=) | [1](https://img.jgi.doe.gov/cgi-bin/mer/main.cgi?section=PhyloProfile&page=phyloProfileGenes&type=func&procId=80526&id=COG1230&taxon_oid=2744055079&bin_oid=) | [1](https://img.jgi.doe.gov/cgi-bin/mer/main.cgi?section=PhyloProfile&page=phyloProfileGenes&type=func&procId=80526&id=COG1230&taxon_oid=2744055078&bin_oid=) | | [1](https://img.jgi.doe.gov/cgi-bin/mer/main.cgi?section=PhyloProfile&page=phyloProfileGenes&type=func&procId=80526&id=COG1230&taxon_oid=2744055076&bin_oid=) | | [1](https://img.jgi.doe.gov/cgi-bin/mer/main.cgi?section=PhyloProfile&page=phyloProfileGenes&type=func&procId=80526&id=COG1230&taxon_oid=2744055077&bin_oid=) | | [1](https://img.jgi.doe.gov/cgi-bin/mer/main.cgi?section=PhyloProfile&page=phyloProfileGenes&type=func&procId=80526&id=COG1230&taxon_oid=2744055075&bin_oid=) | | [1](https://img.jgi.doe.gov/cgi-bin/mer/main.cgi?section=PhyloProfile&page=phyloProfileGenes&type=func&procId=80526&id=COG1230&taxon_oid=2744055074&bin_oid=) | | [1](https://img.jgi.doe.gov/cgi-bin/mer/main.cgi?section=PhyloProfile&page=phyloProfileGenes&type=func&procId=80526&id=COG1230&taxon_oid=2744055073&bin_oid=) | | [1](https://img.jgi.doe.gov/cgi-bin/mer/main.cgi?section=PhyloProfile&page=phyloProfileGenes&type=func&procId=80526&id=COG1230&taxon_oid=2744055072&bin_oid=) | | [1](https://img.jgi.doe.gov/cgi-bin/mer/main.cgi?section=PhyloProfile&page=phyloProfileGenes&type=func&procId=80526&id=COG1230&taxon_oid=2744055071&bin_oid=) | | [1](https://img.jgi.doe.gov/cgi-bin/mer/main.cgi?section=PhyloProfile&page=phyloProfileGenes&type=func&procId=80526&id=COG1230&taxon_oid=2744055070&bin_oid=) | | [1](https://img.jgi.doe.gov/cgi-bin/mer/main.cgi?section=PhyloProfile&page=phyloProfileGenes&type=func&procId=80526&id=COG1230&taxon_oid=2744055069&bin_oid=) | | [1](https://img.jgi.doe.gov/cgi-bin/mer/main.cgi?section=PhyloProfile&page=phyloProfileGenes&type=func&procId=80526&id=COG1230&taxon_oid=2744055068&bin_oid=) | | [1](https://img.jgi.doe.gov/cgi-bin/mer/main.cgi?section=PhyloProfile&page=phyloProfileGenes&type=func&procId=80526&id=COG1230&taxon_oid=2744055067&bin_oid=) | | 1 | |  |
| KO:K10823 | oligopeptide transport system ATP-binding protein (oppF) | beta-Lactam resistance | [1](https://img.jgi.doe.gov/cgi-bin/mer/main.cgi?section=PhyloProfile&page=phyloProfileGenes&type=func&procId=80526&id=COG4300&taxon_oid=2744055081&bin_oid=) | [1](https://img.jgi.doe.gov/cgi-bin/mer/main.cgi?section=PhyloProfile&page=phyloProfileGenes&type=func&procId=80526&id=COG4300&taxon_oid=2744055080&bin_oid=) | [0](https://img.jgi.doe.gov/cgi-bin/mer/main.cgi?section=PhyloProfile&page=phyloProfileGenes&type=func&procId=80526&id=COG4300&taxon_oid=2744055079&bin_oid=) | [1](https://img.jgi.doe.gov/cgi-bin/mer/main.cgi?section=PhyloProfile&page=phyloProfileGenes&type=func&procId=80526&id=COG4300&taxon_oid=2744055078&bin_oid=) | | 1 | | [0](https://img.jgi.doe.gov/cgi-bin/mer/main.cgi?section=PhyloProfile&page=phyloProfileGenes&type=func&procId=80526&id=COG4300&taxon_oid=2744055077&bin_oid=) | | [1](https://img.jgi.doe.gov/cgi-bin/mer/main.cgi?section=PhyloProfile&page=phyloProfileGenes&type=func&procId=80526&id=COG4300&taxon_oid=2744055075&bin_oid=) | | 1 | | [0](https://img.jgi.doe.gov/cgi-bin/mer/main.cgi?section=PhyloProfile&page=phyloProfileGenes&type=func&procId=80526&id=COG4300&taxon_oid=2744055073&bin_oid=) | | [1](https://img.jgi.doe.gov/cgi-bin/mer/main.cgi?section=PhyloProfile&page=phyloProfileGenes&type=func&procId=80526&id=COG4300&taxon_oid=2744055072&bin_oid=) | | 0 | | [0](https://img.jgi.doe.gov/cgi-bin/mer/main.cgi?section=PhyloProfile&page=phyloProfileGenes&type=func&procId=80526&id=COG4300&taxon_oid=2744055070&bin_oid=) | | 0 | | [0](https://img.jgi.doe.gov/cgi-bin/mer/main.cgi?section=PhyloProfile&page=phyloProfileGenes&type=func&procId=80526&id=COG4300&taxon_oid=2744055068&bin_oid=) | | [0](https://img.jgi.doe.gov/cgi-bin/mer/main.cgi?section=PhyloProfile&page=phyloProfileGenes&type=func&procId=80526&id=COG4300&taxon_oid=2744055067&bin_oid=) | | 0 | |  |
|  |  |  | 11 | 14 | 8 | 11 | | 8 | | 8 | | 11 | | 8 | | 8 | | 14 | | 8 | | 10 | | 8 | | 8 | | 8 | | 10 | |  |
| KO:K01775 | alanine racemase [EC:5.1.1.1] (alr) | Vancomycin resistance | 4 | 4 | 4 | 4 | | 4 | | 4 | | 4 | | 4 | | 4 | | 4 | | 5 | | 4 | | 4 | | 5 | | 4 | | 6 | |  |
| KO:K01921 | D-alanine-D-alanine ligase [EC:6.3.2.4] (ddl) | Vancomycin resistance | 2 | 2 | 2 | 2 | | 2 | | 2 | | 2 | | 2 | | 2 | | 2 | | 2 | | 2 | | 2 | | 2 | | 2 | | 2 | |  |
| KO:K01000 | phospho-N-acetylmuramoyl-pentapeptide-transferase [EC:2.7.8.13] (mraY) | Vancomycin resistance | 1 | [1](https://img.jgi.doe.gov/cgi-bin/mer/main.cgi?section=PhyloProfile&page=phyloProfileGenes&type=func&procId=77255&id=COG0798&taxon_oid=2744055080&bin_oid=) | [1](https://img.jgi.doe.gov/cgi-bin/mer/main.cgi?section=PhyloProfile&page=phyloProfileGenes&type=func&procId=77255&id=COG0798&taxon_oid=2744055079&bin_oid=) | [1](https://img.jgi.doe.gov/cgi-bin/mer/main.cgi?section=PhyloProfile&page=phyloProfileGenes&type=func&procId=77255&id=COG0798&taxon_oid=2744055078&bin_oid=) | | [1](https://img.jgi.doe.gov/cgi-bin/mer/main.cgi?section=PhyloProfile&page=phyloProfileGenes&type=func&procId=77255&id=COG0798&taxon_oid=2744055076&bin_oid=) | | [1](https://img.jgi.doe.gov/cgi-bin/mer/main.cgi?section=PhyloProfile&page=phyloProfileGenes&type=func&procId=77255&id=COG0798&taxon_oid=2744055077&bin_oid=) | | [1](https://img.jgi.doe.gov/cgi-bin/mer/main.cgi?section=PhyloProfile&page=phyloProfileGenes&type=func&procId=77255&id=COG0798&taxon_oid=2744055075&bin_oid=) | | [1](https://img.jgi.doe.gov/cgi-bin/mer/main.cgi?section=PhyloProfile&page=phyloProfileGenes&type=func&procId=77255&id=COG0798&taxon_oid=2744055074&bin_oid=) | | 1 | | [1](https://img.jgi.doe.gov/cgi-bin/mer/main.cgi?section=PhyloProfile&page=phyloProfileGenes&type=func&procId=77255&id=COG0798&taxon_oid=2744055072&bin_oid=) | | [1](https://img.jgi.doe.gov/cgi-bin/mer/main.cgi?section=PhyloProfile&page=phyloProfileGenes&type=func&procId=77255&id=COG0798&taxon_oid=2744055071&bin_oid=) | | [1](https://img.jgi.doe.gov/cgi-bin/mer/main.cgi?section=PhyloProfile&page=phyloProfileGenes&type=func&procId=77255&id=COG0798&taxon_oid=2744055070&bin_oid=) | | 1 | | [1](https://img.jgi.doe.gov/cgi-bin/mer/main.cgi?section=PhyloProfile&page=phyloProfileGenes&type=func&procId=77255&id=COG0798&taxon_oid=2744055068&bin_oid=) | | [1](https://img.jgi.doe.gov/cgi-bin/mer/main.cgi?section=PhyloProfile&page=phyloProfileGenes&type=func&procId=77255&id=COG0798&taxon_oid=2744055067&bin_oid=) | | 1 | |  |
| KO:K01929 | UDP-N-acetylmuramoyl-tripeptide--D-alanyl-D-alanine ligase [EC:6.3.2.10] (murF) | Vancomycin resistance | [1](https://img.jgi.doe.gov/cgi-bin/mer/main.cgi?section=PhyloProfile&page=phyloProfileGenes&type=func&procId=77255&id=COG1230&taxon_oid=2744055081&bin_oid=) | [1](https://img.jgi.doe.gov/cgi-bin/mer/main.cgi?section=PhyloProfile&page=phyloProfileGenes&type=func&procId=77255&id=COG1230&taxon_oid=2744055080&bin_oid=) | [1](https://img.jgi.doe.gov/cgi-bin/mer/main.cgi?section=PhyloProfile&page=phyloProfileGenes&type=func&procId=77255&id=COG1230&taxon_oid=2744055079&bin_oid=) | [1](https://img.jgi.doe.gov/cgi-bin/mer/main.cgi?section=PhyloProfile&page=phyloProfileGenes&type=func&procId=77255&id=COG1230&taxon_oid=2744055078&bin_oid=) | | [1](https://img.jgi.doe.gov/cgi-bin/mer/main.cgi?section=PhyloProfile&page=phyloProfileGenes&type=func&procId=77255&id=COG1230&taxon_oid=2744055076&bin_oid=) | | [1](https://img.jgi.doe.gov/cgi-bin/mer/main.cgi?section=PhyloProfile&page=phyloProfileGenes&type=func&procId=77255&id=COG1230&taxon_oid=2744055077&bin_oid=) | | [1](https://img.jgi.doe.gov/cgi-bin/mer/main.cgi?section=PhyloProfile&page=phyloProfileGenes&type=func&procId=77255&id=COG1230&taxon_oid=2744055075&bin_oid=) | | [1](https://img.jgi.doe.gov/cgi-bin/mer/main.cgi?section=PhyloProfile&page=phyloProfileGenes&type=func&procId=77255&id=COG1230&taxon_oid=2744055074&bin_oid=) | | [1](https://img.jgi.doe.gov/cgi-bin/mer/main.cgi?section=PhyloProfile&page=phyloProfileGenes&type=func&procId=77255&id=COG1230&taxon_oid=2744055073&bin_oid=) | | [1](https://img.jgi.doe.gov/cgi-bin/mer/main.cgi?section=PhyloProfile&page=phyloProfileGenes&type=func&procId=77255&id=COG1230&taxon_oid=2744055072&bin_oid=) | | [1](https://img.jgi.doe.gov/cgi-bin/mer/main.cgi?section=PhyloProfile&page=phyloProfileGenes&type=func&procId=77255&id=COG1230&taxon_oid=2744055071&bin_oid=) | | [1](https://img.jgi.doe.gov/cgi-bin/mer/main.cgi?section=PhyloProfile&page=phyloProfileGenes&type=func&procId=77255&id=COG1230&taxon_oid=2744055070&bin_oid=) | | [1](https://img.jgi.doe.gov/cgi-bin/mer/main.cgi?section=PhyloProfile&page=phyloProfileGenes&type=func&procId=77255&id=COG1230&taxon_oid=2744055069&bin_oid=) | | [1](https://img.jgi.doe.gov/cgi-bin/mer/main.cgi?section=PhyloProfile&page=phyloProfileGenes&type=func&procId=77255&id=COG1230&taxon_oid=2744055068&bin_oid=) | | [1](https://img.jgi.doe.gov/cgi-bin/mer/main.cgi?section=PhyloProfile&page=phyloProfileGenes&type=func&procId=77255&id=COG1230&taxon_oid=2744055067&bin_oid=) | | 1 | |  |
| KO:K02563 | UDP-N-acetylglucosamine--N-acetylmuramyl-(pentapeptide) pyrophosphoryl-undecaprenol N-acetylglucosamine transferase [EC:2.4.1.227] (murG) | Vancomycin resistance | [1](https://img.jgi.doe.gov/cgi-bin/mer/main.cgi?section=PhyloProfile&page=phyloProfileGenes&type=func&procId=77255&id=COG1668&taxon_oid=2744055081&bin_oid=) | [1](https://img.jgi.doe.gov/cgi-bin/mer/main.cgi?section=PhyloProfile&page=phyloProfileGenes&type=func&procId=77255&id=COG1668&taxon_oid=2744055080&bin_oid=) | [1](https://img.jgi.doe.gov/cgi-bin/mer/main.cgi?section=PhyloProfile&page=phyloProfileGenes&type=func&procId=77255&id=COG1668&taxon_oid=2744055079&bin_oid=) | [1](https://img.jgi.doe.gov/cgi-bin/mer/main.cgi?section=PhyloProfile&page=phyloProfileGenes&type=func&procId=77255&id=COG1668&taxon_oid=2744055078&bin_oid=) | | [1](https://img.jgi.doe.gov/cgi-bin/mer/main.cgi?section=PhyloProfile&page=phyloProfileGenes&type=func&procId=77255&id=COG1668&taxon_oid=2744055076&bin_oid=) | | [1](https://img.jgi.doe.gov/cgi-bin/mer/main.cgi?section=PhyloProfile&page=phyloProfileGenes&type=func&procId=77255&id=COG1668&taxon_oid=2744055077&bin_oid=) | | [1](https://img.jgi.doe.gov/cgi-bin/mer/main.cgi?section=PhyloProfile&page=phyloProfileGenes&type=func&procId=77255&id=COG1668&taxon_oid=2744055075&bin_oid=) | | [1](https://img.jgi.doe.gov/cgi-bin/mer/main.cgi?section=PhyloProfile&page=phyloProfileGenes&type=func&procId=77255&id=COG1668&taxon_oid=2744055074&bin_oid=) | | [1](https://img.jgi.doe.gov/cgi-bin/mer/main.cgi?section=PhyloProfile&page=phyloProfileGenes&type=func&procId=77255&id=COG1668&taxon_oid=2744055073&bin_oid=) | | [1](https://img.jgi.doe.gov/cgi-bin/mer/main.cgi?section=PhyloProfile&page=phyloProfileGenes&type=func&procId=77255&id=COG1668&taxon_oid=2744055072&bin_oid=) | | 1 | | [1](https://img.jgi.doe.gov/cgi-bin/mer/main.cgi?section=PhyloProfile&page=phyloProfileGenes&type=func&procId=77255&id=COG1668&taxon_oid=2744055070&bin_oid=) | | [1](https://img.jgi.doe.gov/cgi-bin/mer/main.cgi?section=PhyloProfile&page=phyloProfileGenes&type=func&procId=77255&id=COG1668&taxon_oid=2744055069&bin_oid=) | | [1](https://img.jgi.doe.gov/cgi-bin/mer/main.cgi?section=PhyloProfile&page=phyloProfileGenes&type=func&procId=77255&id=COG1668&taxon_oid=2744055068&bin_oid=) | | [1](https://img.jgi.doe.gov/cgi-bin/mer/main.cgi?section=PhyloProfile&page=phyloProfileGenes&type=func&procId=77255&id=COG1668&taxon_oid=2744055067&bin_oid=) | | 1 | |  |
| KO:K07260 | D-alanyl-D-alanine carboxypeptidase [EC:3.4.16.4] (vanY) | Vancomycin resistance | 1 | 1 | 1 | 1 | | 1 | | 1 | | 1 | | 1 | | 1 | | 1 | | 1 | | 1 | | 1 | | 1 | | 1 | | 1 | |  |
| KO:K18351 | two-component system, OmpR family, sensor histidine kinase VanS [EC:2.7.13.3] (vanSAc) | Vancomycin resistance | 1 | 1 | 1 | 1 | | 1 | | 1 | | 1 | | 1 | | 1 | | 1 | | 1 | | 1 | | 1 | | 1 | | 1 | | 1 | |  |
| KO:K18352 | two-component system, OmpR family, response regulator VanR (vanRAc) | Vancomycin resistance | 1 | 1 | 1 | 1 | | 1 | | 1 | | 1 | | 1 | | 1 | | 1 | | 1 | | 1 | | 1 | | 1 | | 1 | | 1 | |  |
| KO:K18353 | vancomycin resistance protein VanJ (vanJ) | Vancomycin resistance | 1 | 1 | 1 | 1 | | 1 | | 1 | | 1 | | 1 | | 1 | | 1 | | 0 | | 0 | | 1 | | 0 | | 1 | | 0 | |  |
|  |  |  | 13 | 13 | 13 | 13 | | 13 | | 13 | | 13 | | 13 | | 13 | | 13 | | 13 | | 12 | | 13 | | 13 | | 13 | | 14 | |  |
| KO:K05786 | chloramphenicol-sensitive protein RarD (rarD) | Chloramphenicol resistance | 1 | 1 | 1 | 1 | | 1 | | 1 | | 1 | | 1 | | 1 | | 1 | | 1 | | 1 | | 1 | | 1 | | 1 | | 1 | |  |
| KO:K07552 | MFS transporter, DHA1 family, bicyclomycin/chloramphenicol resistance protein (bcr) | Chloramphenicol resistance | 2 | 2 | 2 | 2 | | 2 | | 2 | | 2 | | 2 | | 2 | | 2 | | 2 | | 2 | | 2 | | 2 | | 2 | | 1 | |  |
| KO:K18553 | MFS transporter, DHA1 family, chloramphenicol resistance protein (cmlR, cmx) | Chloramphenicol resistance | 1 | 1 | 1 | 1 | | 1 | | 1 | | 1 | | 1 | | 1 | | 1 | | 1 | | 1 | | 1 | | 1 | | 1 | | 0 | |  |
|  |  |  |  |  |  |  | |  | |  | |  | |  | |  | |  | |  | |  | |  | |  | |  | |  | |  |
| COG0431 | Chromate reductase (chrR) | NAD(P)H-dependent FMN reductase | 1 | 1 | 1 | 1 | | 2 | | 1 | | 1 | | 2 | | 1 | | 1 | | 1 | | 1 | | 1 | | 1 | | 1 | | 1 | |  |
| COG4300 | Cadmium resistance protein CadD, predicted permease | Inorganic ion transport and metabolism | 1 | 1 | 1 | 1 | | 1 | | 0 | | 1 | | 1 | | 0 | | 1 | | 1 | | 0 | | 1 | | 0 | | 1 | | 1 | |  |
| COG1230 | Co/Zn/Cd efflux system component | Inorganic ion transport and metabolism | 2 | 2 | 2 | 2 | | 2 | | 1 | | 2 | | 2 | | 1 | | 2 | | 2 | | 1 | | 2 | | 2 | | 2 | | 2 | |  |
| COG0798 | Arsenite efflux pump ArsB, ACR3 family | Inorganic ion transport and metabolism | 1 | 0 | 2 | 1 | | 2 | | 1 | | 1 | | 1 | | 1 | | 0 | | 1 | | 1 | | 2 | | 0 | | 2 | | 1 | |  |
